# Supplementary material for: Calcium Superphosphate-Mediated Reshaping of Denitrifying Bacteria Community Contributed to N2O Mitigation in Pig Manure Windrow Composting
Source: Int J Environ Res Public Health. 2020 Dec 29;18(1):171. doi: 10.3390/ijerph18010171 (PMC7795020; doi:10.3390/ijerph18010171)

Article

Calcium Superphosphate-Mediated Reshaping of Denitrifying Bacteria Community Contributed to N_2O Mitigation in Pig Manure Windrow Composting

Yaguo Jin [1], Yingcheng Miao [1], Yajun Geng [1], Mengyuan Huang [1], Yihe Zhang [1], Xiuchao Song [2] and Shuqing Li [1,3,*] and Jianwen Zou [1,3]

[1] Jiangsu Key Laboratory of Low Carbon Agriculture and GHGs Mitigation, College of Resources and Environmental Sciences, Nanjing Agricultural University, Nanjing 210095, China; 2015203017@njau.edu.cn (Y.J.); 2017103088@njau.edu.cn (Y.M.); 2017203042@njau.edu.cn (Y.G.); 2018103046@njau.edu.cn (M.H.); 2020203073@stu.njau.edu.cn (Y.Z.); jwzou21@njau.edu.cn (J.Z.)

[2] Institute of Agricultural Resources and Environment, Jiangsu Academy of Agricultural Sciences, Nanjing 210014, China; xiuchao103@163.com

[3] Jiangsu Key Lab and Engineering Center for Solid Organic Waste Utilization, Jiangsu Collaborative Innovation Center for Solid Organic Waste Resource Utilization, Nanjing Agricultural University, Nanjing 210095, China

* Correspondence: shuqingli@njau.edu.cn; Tel.: +86-25-8439-6286; Fax: +86-25-8439-5210

Citation: Jin, Y.; Miao, Y; Geng, Y; Huang, M; Zhang, Y; Song, X; Li, S; Zou, J. Calcium Superphosphate-Mediated Reshaping of Denitrifying Bacteria Community Contributed to N_2O Mitigation in Pig Manure Windrow Composting. *Int. J. Environ. Res. Public Health* **2021**, *18*, 171. https://doi.org/10.3390/ijerph18010171

Received: 1 December 2020
Accepted: 23 December 2020
Published: 29 December 2020

Publisher's Note: MDPI stays neutral with regard to jurisdictional claims in published maps and institutional affiliations.

Copyright: © 2020 by the authors. Submitted for possible open access publication under the terms and conditions of the Creative Commons Attribution (CC BY) license (http://creativecommons.org/licenses/by/4.0/).

To be submitted to *International Journal of Environmental Research and Public Health*

Figure S1. The pictures of the experimental static chamber system for gas sampling. (A) The PVC chamber bases (30 cm length × 30 cm width × 25 cm height) were inserted 25 cm into the pile at 10–12 h before gas sampling. (B) The opaque chamber (diameter 0.45 m, height 5 m) was placed on the peak of each windrow with rim of the chamber fitted into the groove of collar. As sampling, the groove in the top edge of the collar was filled with water to seal the rim of the chamber.

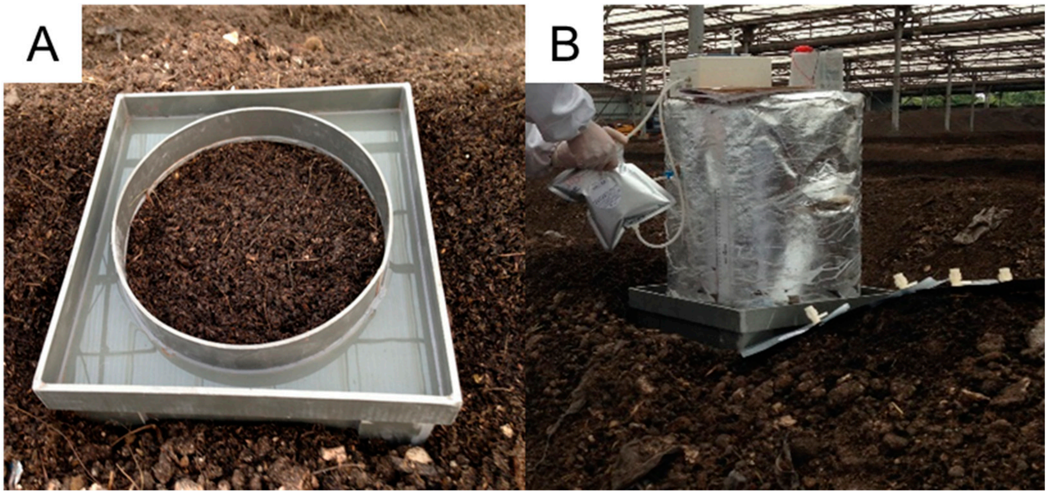

Figure S2. Accumulative N_2O emission over the total composting period in Control and calcium superphosphate-added (CaSSP) piles.

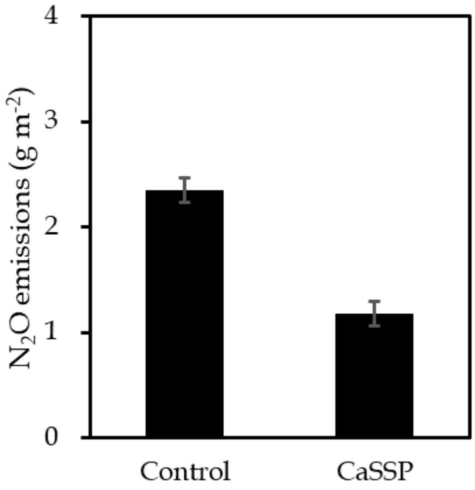

Supplement: Supplementary file 1 [file ijerph-18-00171-s001.pdf]
